# Jumping for Joy: The Importance of the Body and of Dynamics in the Expression and Recognition of Positive Emotions

**DOI:** 10.3389/fpsyg.2018.00763

**Published:** 2018-05-15

**Authors:** Marcello Mortillaro, Daniel Dukes

**Affiliations:** ^1^Swiss Center for Affective Sciences, University of Geneva, Geneva, Switzerland; ^2^Psychology Research Institute, University of Amsterdam, Amsterdam, Netherlands

**Keywords:** emotion, positive emotions, dynamics, facial expression, bodily expression, emotion expression, emotion recognition

## Abstract

The majority of research on emotion expression has focused on static facial prototypes of a few selected, mostly negative emotions. Implicitly, most researchers seem to have considered all positive emotions as sharing one common signal (namely, the smile), and consequently as being largely indistinguishable from each other in terms of expression. Recently, a new wave of studies has started to challenge the traditional assumption by considering the role of multiple modalities and the dynamics in the expression and recognition of positive emotions. Based on these recent studies, we suggest that positive emotions are better expressed and correctly perceived when (a) they are communicated simultaneously through the face and body and (b) perceivers have access to dynamic stimuli. Notably, we argue that this improvement is comparatively more important for positive emotions than for negative emotions. Our view is that the misperception of positive emotions has fewer immediate and potentially life-threatening consequences than the misperception of negative emotions; therefore, from an evolutionary perspective, there was only limited benefit in the development of clear, quick signals that allow observers to draw fine distinctions between them. Consequently, we suggest that the successful communication of positive emotions requires a stronger signal than that of negative emotions, and that this signal is provided by the use of the body and the way those movements unfold. We hope our contribution to this growing field provides a new direction and a theoretical grounding for the many lines of empirical research on the expression and recognition of positive emotions.

## Introduction

The last 15 years have seen unprecedented interest in positive emotions, sustained, presumably, by the development of fields like positive psychology (Fredrickson and Joiner, 2002) and emotional intelligence (Quoidbach et al., 2010; Nelis et al., 2011). Before then, emotion research had largely focused on a set of almost entirely negative emotions that had been identified by Ekman (1992, 1993). In fact, Ekman’s original set of basic emotions featured only one positive emotion – joy or happiness – and, consequently, several authors considered joy-happiness as the *only* positive emotion in their early studies (e.g., Oatley and Johnson-Laird, 1987). Conceiving of positive emotion in this way led to them being treated as one, single, undifferentiated class of events, and this naturally became an obstacle toward progress in positive emotion research. In perhaps the clearest sign that the field has since matured, a recent and comprehensive review by Shiota et al. (2017) argues that positive emotions may be differentiated based on distinct autonomic nervous system signatures, different effects on cognition and judgment, and specific non-verbal behaviors.

In this article, we focus on the non-verbal behaviors associated with positive emotions. We offer a new perspective as to why the quest for the identification of specific signals of positive emotions needs to be redirected beyond static prototypical faces. We are aware that the positive vs. negative distinction could be debated and that emotional communication is a more complex process than the simple perception of emotion categories – as we have discussed elsewhere (Mortillaro et al., 2013; Scherer et al., 2013, 2018; Reschke et al., 2017). However, this paper is about the signals that can be used for the accurate communication of pleasant emotional states (e.g., a smile that signals embarrassment is not one of them) and does not assume that these signals are exclusive to genuine emotion signaling (a polite smile is a pure social signal). The reader should be aware that this is a brief perspective paper and not an attempt at an exhaustive review. We therefore focus on the most relevant literature for our argument and highlight what is novel and worthwhile about our perspective. Furthermore, we decided to focus on why this quest should include the dynamics of facial movements and the body, although a similar case could be made to include the voice (Sauter and Scott, 2007; Sauter, 2017), the context (Hassin et al., 2013; Aviezer et al., 2017), and even autonomic signals like pupil dilation (Kret, 2015).

We begin with an overview of the standard accounts of facial emotion expression and recognition, before providing a justification for why we feel a change in direction for empirical studies of positive emotion is necessary.

## Enjoyment Smile: The Only Sign for All Positive Emotions?

Research in non-verbal behavior in emotion has traditionally concentrated on the face and, following the approach used by Ekman to identify basic emotions, has aimed at identifying prototypical configurations of facial expression. However, this approach has not proved very successful for positive emotions.

Progress was initially hampered by an implicit consensus that all positive emotions were essentially expressed in the same way. Notably, the enjoyment smile [the result of the action of the *zygomaticus major* muscle and the contraction of the *orbicularis oculi pars lateralis* muscle (Ekman and Friesen, 1978)] was originally held to be the only (and ubiquitous) sign of positive emotions. In a quote from 1992 that not only outlines the problem but also offers a possible solution, Ekman wrote, “One of the questions remaining about smiles is whether the different positive emotions (e.g., amusement, contentment, relief, etc.) have distinctive forms of smiling, or if the variety of positive emotions share one signal and can be inferred only from other behavioral or contextual cues. I presume that all of these forms of enjoyment share the musculature described by Duchenne, and are distinguished by their dynamics, not their morphology” (Ekman, 1992, p. 67).

Several studies have since then shown that there are various types of smiles, with different interpersonal functions (for example, Rychlowska et al., 2017), and that most smiles are social signals and not simple reflections of inner feelings (Fridlund, 1997). However, even when signs other than the smile are included, the pool of positive emotions linked to particular static expressions remains very limited, and there are only a few studies that have explicitly compared multiple positive emotion expressions (e.g., Hofmann et al., 2017). In one notable exception, Campos et al. (2013) confirmed the critical role of the Duchenne smile across several positive emotions. The authors identified associations between each positive emotion and some facial action units, but the resulting configurations were not entirely different while it was the inclusion of head and upper body movements that made the emotions more distinguishable. For example, facial expressions of pride and contentment can be differentiated only by their associated head position.

In a recent review, Sauter (2017) suggests a more complex version of Ekman’s view of positive emotion as a family of ‘forms of enjoyment.’ In fact, Sauter suggests four families of positive emotions – ‘epistemological,’ ‘prosocial,’ ‘savoring,’ and ‘agency-approach.’ Based on her review, only epistemological emotions (amusement, awe, interest, and relief) and pride appear to have distinct recognizable facial and/or vocal displays. It is worth noting, however, that the prototypical expression of pride also includes bodily movements aimed at postural expansion, which involves, for example, pulling the shoulders back and raising the head.

All in all, there is only weak evidence for the differentiation between positive emotions based on static facial features. We hypothesize that the expressive elements that differentiate positive emotions most clearly reside in the dynamics of facial expression and in the body.

## Hypothesis: Facial Dynamics and Body Representations are Critical for Distinguishing Non-Verbal Displays of Positive Emotion

From a functional perspective, there is an enduring debate about whether emotion expressions are direct reflections of inner-states (I smile because I am happy), or whether emotions are expressed as social signals (I smile at you to show you I am happy; see Parkinson, 2005). From an evolutionary perspective, this debate is often drawn along the lines of whether the emotional expression is made for the benefits of the expresser (such as when someone widens his/her eyes in states of fear to increase the perceptual uptake in order to prepare his/her escape from danger) which may serve as an emotional cue to observers, or, alternatively, whether the expression may be used intentionally to communicate something to observers (for a discussion, see Schmidt and Cohn, 2001; Kret and Straffon, 2018).

In order to demonstrate our argument, we will focus on what the observer picks up from the expression rather than the processes that produce the expression (Frijda and Tcherkassof, 1997). In evolutionary terms, negative emotions (e.g., fear and anger) are more critical for survival than positive emotions (e.g., pride and interest) because they are more likely to be understood as signs of potentially life-threatening situations that require an immediate response. There is an element of urgency that is not present in the case of positive emotions and that requires the signal to be understood quickly, clearly, and very specifically. These are the benefits of prototypical facial expressions; they have a “snapshot” quality that makes them rapidly recognizable and the emotions effectively identifiable (Ekman, 1993). Consequently, it makes sense that signals have evolved to rapidly and effectively communicate the potential dangers in the environment to conspecifics and that skills have evolved to recognize that threat. In a recent study, Gold et al. (2013) found that participants could recognize the traditional six basic emotions (including joy as the only positive emotion) with comparable accuracy regardless of whether they viewed the expressions as naturally evolving, temporally reversed, temporally randomized expressions, or as a single snapshot. This result supports the hypothesis that dynamic information is not necessary for the correct recognition of basic negative emotions.

The fact the positive emotions are less critical for survival is not to deny the importance of their social functions. Positive emotions are involved in affiliation and cooperation and therefore important for adaptation (Campos et al., 2015). Different positive emotions have specific functions – respond to material opportunities or social stimuli, facilitate playing new skills, encode novel information – that require distinct expressive signals to be effectively communicated (Shiota et al., 2014). However, as mentioned previously, it appears that static faces do not provide a clear enough signal. While static facial expressions are sufficient for distinguishing negative emotions in most circumstances, we argue that the distinction between positive emotions critically requires additional information that is provided by the dynamics and body representations.

Dynamic representations of emotion expressions evidently contain more information than static ones, but they do not always increase the rate at which emotions are recognized (Scherer et al., 2011). In fact, it is not the sum of static cues that explains why dynamic stimuli are better recognized in some conditions, but rather the specific information that is conveyed by the movement (Ambadar et al., 2005). Interestingly, Jack et al. (2014) suggest that the perception process is temporally driven and that dynamic facial expressions transmit an evolving hierarchy of signals over time, from biologically basic (approach/avoidance) to social information, such as emotion categories. Similarly, the increase in information provided by adding bodily information to facial expressions does not automatically increase the rate at which emotions are correctly recognized. Studies show that the interaction between bodies and faces is more complex than simply aggregating the information from each modality (Aviezer et al., 2008, 2012).

App et al. (2011) suggest that the body promotes social-status emotions, that the face promotes survival emotions, and that touch promotes intimate emotions. Elsewhere, Martinez et al. (2016) found that for the standard set of six basic emotions, five of which are negative, the face was significantly better than the body in conveying emotional information. Again, these two studies provide indirect support for our hypothesis that the face is critical and sufficient for the communication of basic, survival-related emotions, but not for other types of emotions.

It seems then that good evolutionary, social and functional justifications can be found for arguing that positive emotions need to be signaled more “loudly” in order to be correctly identified and recognized than negative emotions. We turn now to recent empirical studies that seem to support our argument.

### Evidence About Dynamic Facial Expressions of Positive Emotions

Researchers mostly used – and still use – static prototypical facial expressions in their studies (Scherer et al., 2011). Recently, however, there is a growing trend toward the use of dynamic expressions that do not fully correspond to the traditional prototypes (Bänziger et al., 2012; O’Reilly et al., 2016; Krumhuber et al., 2017). This methodological choice allows emotions to be studied that are not found in the standard basic set (as there is no fixed, pre-defined prototype to be portrayed) and to compare subtly different emotions.

In a recent review concerning the role of dynamics in emotion recognition, Krumhuber et al. (2013, p. 42) wrote that motion “…confers particular benefits when static information is inefficient or unavailable.” Given the absence of prototypical facial configurations, it is therefore not surprising that the study of positive emotions has benefited from the inclusion of dynamic stimuli. Indeed, movement dynamics are an integral part of the emotion perception process, and it is used by perceivers to differentiate deliberate and genuine smiles (that is when the smiles are spontaneous and reflect a felt positive emotional state) or to judge the naturalness of the emotion expression tout court (Sato and Yoshikawa, 2004; Krumhuber and Kappas, 2005; Schmidt et al., 2006). In one pioneering study using synthetic facial expressions, Wehrle et al. (2000) and Kaiser and Wehrle (2001) found that positive emotional states such as pleasure, happiness, and elation, could be distinguished by their facial expressions when dynamic stimuli were presented. In a more recent study, Mortillaro et al. (2011) showed that joy, interest, pride, and sensory pleasure could only be distinguished when the dynamic properties of the expressions were taken into account. It was not the presence or the absence of certain facial movements that could be used to reliably differentiate these emotions, but rather the duration of the movements and their frequency within one emotion expression. Similarly, Fujimura and Suzuki (2010) found that two out of the three positive emotions that they included in their study were significantly better recognized in the dynamic than in the static presentation mode, while only one out of the five non-positive emotions (fearful) showed the same significant advantage when presented dynamically.

Other studies have demonstrated the special role of dynamic movements for specific positive emotions. For example, while the search for a prototypical static facial expression of interest has proven inconclusive, emotional expressions of interest can be well recognized when it presented in a dynamic fashion (Dukes et al., 2017). Furthermore, Nelson and Russell (2014) have shown that different types of pride can only be differentiated when dynamically presented. Similarly, Namba et al. (2017) found a different dynamic pattern of movements in posed and spontaneous expressions of amusement – a difference that did not appear in static expressions.

Overall, it appears that the dynamic representation of positive emotions may be critical for them to be readily identified and differentiated (for a similar position, see Fujimura et al., 2012).

### Evidence About the Bodily Expression of Positive Emotions

The expression of emotions through body movements and gestures has been understudied in comparison to facial and vocal expressions [for a general discussion of the neurological basis of the perception of emotions from the body and for the reasons to consider bodily expressions in affective science, please see the works of de Gelder (2006, 2009)]. Nevertheless, results of a number of studies showed that emotions can be recognized from bodies (e.g., de Gelder and Van den Stock, 2011) and even from very limited information like point-light body displays (Atkinson et al., 2004). A full review of this literature is beyond the scope of a perspective article and therefore, we will only discuss studies that investigated the bodily expression of several positive emotions.

In one of the largest studies available on the bodily expression of emotions, Dael et al. (2012) identified patterns of body movements that were specific to positive emotions. Even more importantly, they showed that positive emotions could be correctly discriminated from their bodily movements alone, even more so than the negative emotions. On average the positive emotions were correctly classified 63.3% of the time on the basis of bodily movements (when chance level was 8.33%), while the negative emotions were only correctly classified 46.7% of the time.

Similarly, App et al. (2011) found that pride and love were better recognized in the body than in the face, while happiness and sympathy were recognized at the same level in the two modalities. Dael et al. (2013) studied the dynamic properties of arm movements. Even though they did not explicitly compare the six positive emotions, substantial differences among them are clear in most, if not all, the parameters they reported. This corroborates our hypothesis that bodily movements are critical for distinguishing between positive emotions.

The effects of bodily representations on expressing specific positive emotions also tend to support our argument. The clearest case comes from research on pride for which there is general consensus about a prototypical expression involving a particular posture and specific gestures (Tracy and Robins, 2004). Another positive emotion for which the body seems to carry important information is interest. Dukes et al. (2017) found that facial expressions alone were not able to reliably communicate interest; however, when the face was paired with the body, the recognition accuracy for interest more than doubled, and interest became as easily recognized as Ekman’s six basic emotions.

There is sufficient empirical evidence to suggest that the identification and recognition of positive emotions is made comparatively easier by the inclusion of bodily representations whereas, similarly to the inclusion of dynamic information, this seems less important for negative emotions.

## Conclusion

In this paper, we briefly reviewed some of the most recent and relevant literature on the expression of positive emotions. The results consistently indicate that the research of purely facial static prototypes is likely inconclusive. If specific (or typical) expressions for positive emotions exist, they are more likely to be found in expressions that include dynamic and bodily elements, like body posture and gesture. It is more than 10 years since the prototypical expressions of pride were established and, so far, only a few scholars have pointed out that it is the body and posture or the dynamic representation of these expressions that sets them apart from those of joy. It is now time to accept that static facial expressions are useful, but that they do not capture the whole richness of real-life emotion communication. Future studies, especially when positive emotions are considered, should only use multimodal, dynamic expressions.

## Author Contributions

MM conceived and wrote the first draft of the manuscript. DD contributed to the conception of the article and helped revise the first and subsequent drafts.

## Conflict of Interest Statement

The authors declare that the research was conducted in the absence of any commercial or financial relationships that could be construed as a potential conflict of interest. The reviewer NL and handling Editor declared their shared affiliation.

## References

[B1] Ambadar Z. Schooler J. W. Conn J. F. (2005). Deciphering the enigmatic face the importance of facial dynamics in interpreting subtle facial expressions. 16 403–410. 10.1111/j.0956-7976.2005.01548.x 15869701

[B2] App B. McIntosh D. N. Reed C. L. Hertenstein M. J. (2011). Nonverbal channel use in communication of emotion: how may depend on why. 11 603–617. 10.1037/a0023164 21668111

[B3] Atkinson A. P. Dittrich W. H. Gemmell A. J. Young A. W. (2004). Emotion perception from dynamic and static body expressions in point-light and full-light displays. 33 717–746. 10.1068/p5096 15330366

[B4] Aviezer H. Ensenberg N. Hassin R. R. (2017). The inherently contextualized nature of facial emotion perception. 17 47–54. 10.1016/j.copsyc.2017.06.006 28950972

[B5] Aviezer H. Hassin R. R. Ryan J. Grady C. Susskind J. Anderson A. (2008). Angry, disgusted, or afraid? Studies on the malleability of emotion perception: research article. 19 724–732. 10.1111/j.1467-9280.2008.02148.x 18727789

[B6] Aviezer H. Trope Y. Todorov A. (2012). Body cues, not facial expressions, discriminate between intense positive and negative emotions. 338 1225–1229. 10.1126/science.1224313 23197536

[B7] Bänziger T. Mortillaro M. Scherer K. R. (2012). Introducing the Geneva Multimodal expression corpus for experimental research on emotion perception. 12 1161–1179. 10.1037/a0025827 22081890

[B8] Campos B. Schoebi D. Gonzaga G. C. Gable S. L. Keltner D. (2015). Attuned to the positive? Awareness and responsiveness to others’ positive emotion experience and display. 39 780–794. 10.1007/s11031-015-9494-x

[B9] Campos B. Shiota M. N. Keltner D. Gonzaga G. C. Goetz J. L. (2013). What is shared, what is different? Core relational themes and expressive displays of eight positive emotions. 27 37–52. 10.1080/02699931.2012.683852 22716231

[B10] Dael N. Goudbeek M. Scherer K. R. (2013). Perceived gesture dynamics in nonverbal expression of emotion. 42 642–657. 10.1068/p7364 24422246

[B11] Dael N. Mortillaro M. Scherer K. R. (2012). Emotion expression in body action and posture. 12 1085–1101. 10.1037/a0025737 22059517

[B12] de Gelder B. (2006). Towards the neurobiology of emotional body language. 7 242–249. 10.1038/nrn1872 16495945

[B13] de Gelder B. (2009). Why bodies? Twelve reasons for including bodily expressions in affective neuroscience. 364 3475–3484. 10.1098/rstb.2009.0190 19884142 PMC2781896

[B14] de Gelder B. Van den Stock J. (2011). The bodily expressive action stimulus test (BEAST). Construction and validation of a stimulus basis for measuring perception of whole body expression of emotions. 2:181. 10.3389/fpsyg.2011.00181 21886632 PMC3152787

[B15] Dukes D. Clément F. Audrin C. Mortillaro M. (2017). Looking beyond the static face in emotion recognition: the informative case of interest. 25 575–588. 10.1080/13506285.2017.1341441

[B16] Ekman P. (1992). An argument for basic emotions. 6 169–200. 10.1016/j.concog.2017.10.008 29100796

[B17] Ekman P. (1993). Facial expression and emotion. 48 384–392. 10.1037/0003-066X.48.4.384 8512154

[B18] Ekman P. Friesen W. V. (1978). Palo Alto, CA: Consulting Psychologist Press.

[B19] Fredrickson B. L. Joiner T. (2002). Positive emotions trigger upward spirals toward emotional well-being. 13 172–175. 10.1111/1467-9280.00431 11934003

[B20] Fridlund A. J. (1997). “The new ethology of human facial expressions,” in eds Russell J. A. Dols J. M. F. (Cambridge: Cambridge University Press) 103–129.

[B21] Frijda N. H. Tcherkassof A. (1997). “Facial expressions as modes of action readiness,” in eds Russell J. A. Fernandez-Dols J. M. (Cambridge: Cambridge University Press) 78–102. 10.1017/CBO9780511659911.006

[B22] Fujimura T. Sato W. Okanoya K. (2012). Subcategories of positive emotion. 55 1–8. 10.2117/psysoc.2012.1

[B23] Fujimura T. Suzuki N. (2010). Effects of dynamic information in recognising facial expressions on dimensional and categorical judgments. 39 543–552. 10.1068/p6257 20515001

[B24] Gold J. M. Barker J. D. Barr S. Bittner J. L. Bromfield W. D. Chu N. (2013). The efficiency of dynamic and static facial expression recognition. 13:23. 10.1167/13.5.23. 23620533 PMC3666543

[B25] Hassin R. R. Aviezer H. Bentin S. (2013). Inherently ambiguous: facial expressions of emotions, in context. 5 60–65. 10.1177/1754073912451331 23837206

[B26] Hofmann J. Platt T. Ruch W. (2017). Laughter and smiling in 16 positive emotions. 8 495–507. 10.1109/TAFFC.2017.2737000

[B27] Jack R. E. Garrod O. G. Schyns P. G. (2014). Dynamic facial expressions of emotion transmit an evolving hierarchy of signals over time. 24 187–192. 10.1016/j.cub.2013.11.064 24388852

[B28] Kaiser S. Wehrle T. (2001). “Facial expressions as indicators of appraisal processes,” in eds Scherer K. R. Schorr A. Johnstone T. (New York, NY: Oxford University Press) 285–300.

[B29] Kret M. E. (2015). Emotional expressions beyond facial muscle actions. A call for studying autonomic signals and their impact on social perception. 6:711. 10.3389/fpsyg.2015.00711 26074855 PMC4443639

[B30] Kret M. E. Straffon L. M. (2018). Reply to Crivelli: the different faces of fear and threat. Evolutionary and cultural insights. 10.1016/j.jhevol.2017.11.006 [Epub ahead of print]. 29338887

[B31] Krumhuber E. Kappas A. (2005). Moving smiles: the role of dynamic components for the perception of the genuineness of smiles. 29 3–24. 10.1007/s10919-004-0887-x

[B32] Krumhuber E. Skora P. Küster D. Fou L. (2017). A review of dynamic datasets for facial expression research. 9 280–292. 10.1177/1754073916670022

[B33] Krumhuber E. G. Kappas A. Manstead A. S. R. (2013). Effects of dynamic aspects of facial expressions: a review. 5 41–46. 10.1177/1754073912451349

[B34] Martinez L. Falvello V. B. Aviezer H. Todorov A. (2016). Contributions of facial expressions and body language to the rapid perception of dynamic emotions. 30 939–952. 10.1080/02699931.2015.1035229 25964985

[B35] Mortillaro M. Mehu M. Scherer K. R. (2011). Subtly different positive emotions can be distinguished by their facial expressions. 2 262–271. 10.1177/1948550610389080

[B36] Mortillaro M. Mehu M. Scherer K. R. (2013). “The evolutionary origin of multimodal synchronization and emotional expression,” in eds Altenmüller E. Schmidt S. Zimmermann E. (New York, NY: Oxford University Press) 3–25.

[B37] Namba S. Makihara S. Kabir R. S. Miyatani M. Nakao T. (2017). Spontaneous facial expressions are different from posed facial expressions: morphological properties and dynamic sequences. 36 593–605. 10.1007/s12144-016-9448-9

[B38] Nelis D. Kotsou I. Quoidbach J. Hansenne M. Weytens F. Dupuis P. (2011). Increasing emotional competence improves psychological and physical well-being, social relationships, and employability. 11 354–366. 10.1037/a0021554 21500904

[B39] Nelson N. L. Russell J. A. (2014). Dynamic facial expressions allow differentiation of displays intended to convey positive and hubristic pride. 14 857–864. 10.1037/a0036789 24866524

[B40] Oatley K. Johnson-Laird P. N. (1987). Towards a cognitive theory of emotions. 1 29–50. 10.1080/02699938708408362

[B41] O’Reilly H. Pigat D. Fridenson S. Berggren S. Tal S. Golan O. (2016). The EU-emotion stimulus set: a validation study. 48 567–576. 10.3758/s13428-015-0601-4 26424443

[B42] Parkinson B. (2005). Do facial movements express emotions or communicate motives? 9 278–311. 10.1207/s15327957pspr0904_1 16223353

[B43] Quoidbach J. Berry E. V. Hansenne M. Mikolajczak M. (2010). Positive emotion regulation and well-being: comparing the impact of eight savoring and dampening strategies. 49 368–373. 10.1016/j.paid.2010.03.048

[B44] Reschke P. R. Walle E. A. Dukes D. (2017). Interpersonal development in infancy: the interconnectedness of emotion understanding and social cognition. 11 173–183. 10.1111/cdep.12230

[B45] Rychlowska M. Jack R. E. Garrod O. G. B. Schyns P. G. Martin J. D. Niedenthal P. M. (2017). Functional smiles: tools for love, sympathy, and war. 28 1259–1270. 10.1177/0956797617706082 28741981 PMC6056899

[B46] Sato W. Yoshikawa S. (2004). The dynamic aspects of emotional facial expressions. 18 701–710. 10.1080/02699930341000176

[B47] Sauter D. A. (2017). The nonverbal communication of positive emotions: an emotion family approach. 9 222–234. 10.1177/1754073916667236 28804510 PMC5542129

[B48] Sauter D. A. Scott S. K. (2007). More than one kind of happiness: Can we recognize vocal expressions of different positive states? 31 192–199. 10.1007/s11031-007-9065-x

[B49] Scherer K. R. Clark-Polner E. Mortillaro M. (2011). In the eye of the beholder? Universality and cultural specificity in the expression and perception of emotion. 46 401–435. 10.1080/00207594.2011.626049 22126090

[B50] Scherer K. R. Mortillaro M. Mehu M. (2013). Understanding the mechanisms underlying the production of facial expression of emotion: a componential perspective. 5 47–53. 10.1177/1754073912451504

[B51] Scherer K. R. Mortillaro M. Rotondi I. Sergi I. Trznadel S. (2018). Appraisal-driven facial actions as building blocks for emotion inference. 114 358–379. 10.1037/pspa0000107 29461080

[B52] Schmidt K. L. Ambadar Z. Cohn J. F. Reed L. I. (2006). Movement differences between deliberate and spontaneous facial expressions: zygomaticus major action in smiling. 30 37–52. 10.1007/s10919-005-0003-x 19367343 PMC2668537

[B53] Schmidt K. L. Cohn J. F. (2001). Human facial expressions as adaptations: evolutionary questions in facial expression research. 116 3–24. 10.1002/ajpa.20001 11786989 PMC2238342

[B54] Shiota M. N. Campos B. Oveis C. Hertenstein M. J. Simon-Thomas E. Keltner D. (2017). Beyond happiness: building a science of discrete positive emotions. 72 617–643. 10.1037/a0040456 29016167

[B55] Shiota M. N. Neufeld S. L. Danvers A. F. Osborne E. A. Sng O. Yee C. I. (2014). Positive emotion differentiation: a functional approach. 8 104–117. 10.1111/spc3.12092

[B56] Tracy J. L. Robins R. W. (2004). Show your pride: evidence for a discrete emotion expression. 15 194–197. 10.1111/j.0956-7976.2004.01503008.x 15016291

[B57] Wehrle T. Kaiser S. Schmidt S. Scherer K. R. (2000). Studying the dynamics of emotional expression using synthesized facial muscle movements. 78 105–119. 10.1037//0022-3514.78.1.105 10653509

